# The perception paradox: experiencing and witnessing skin color discrimination and self-rated health in Latin America

**DOI:** 10.3389/fsoc.2026.1816155

**Published:** 2026-08-20

**Authors:** Angela R. Dixon

**Affiliations:** 1 School of Education and Social Policy, Northwestern University, Evanston, IL, United States; 2 Northwestern University Pritzker School of Law, Chicago, IL, United States; 3 Department of Medical Social Sciences, Northwestern University Feinberg School of Medicine, Chicago, IL, United States

**Keywords:** class discrimination, colorism, Latin America, perceived discrimination, self-rated health, vicarious discrimination

## Abstract

**Introduction:**

An extensive body of literature has established that perceived discrimination is linked to adverse health outcomes. In Latin America, a region deemed a pigmentocracy due to its pronounced skin color–based stratification, research on the relationship between color discrimination and health remains limited and mixed. This ambiguity may reflect a perception paradox in the context of national ideologies of *mestizaje* (race-mixture) and racial democracy that purport colorblindness. Latin Americans tend to report high levels of witnessing color discrimination against others (vicarious discrimination) but paradoxically low levels of experiencing discrimination against themselves (personal discrimination).

**Methods:**

Using cross-sectional survey data for Brazil, Mexico, Peru, and Colombia from the Project on Ethnicity and Race in Latin America, I examine the relationship between self-rated health and both personal and vicarious color discrimination. Given that national ideologies have historically privileged class-based explanations of stratification while obscuring the role of skin color, I also examine class discrimination as a comparator.

**Results:**

The results show that the observed associations between discrimination and health vary by both the target and the attribute of discrimination. While personal color discrimination does not show a statistically significant association with poor health, vicarious color discrimination is robustly associated with poor health. In contrast, personal class discrimination is consistently associated with poor health, while vicarious class discrimination is not.

**Discussion:**

Taken together, these findings suggest that associations between perceived discrimination and health must be understood in light of the Latin American racial order in which *mestizaje* and racial democracy work to define which explanations of inequality and unfair treatment are deemed legitimate and reportable.

## 1 Introduction

Perceptions of discrimination are an important measure of how individuals interpret and experience inequality. In societies marked by colorism, which refers to the systematic privileging of white/light skin and devaluing of darker skin, skin color can function as a salient attribute of perceived discrimination (Dixon and Telles, 2017). An extensive body of literature demonstrates that perceived discrimination is associated with adverse mental and physical health (Lam et al., 2025; Paradies et al., 2015; Williams et al., 2019). These studies, primarily based in the U.S. and other high-income countries, have largely focused on personal discrimination, defined as unfair treatment targeted against oneself (Lee et al., 2024; Lewis et al., 2015; Pascoe and Richman, 2009). Considerably less scholarly attention has focused on how different approaches to measuring color discrimination shape our understanding of its relationship to health.

Measurement considerations are particularly important in Latin America given the entrenched color-based stratification that coexists with historic national narratives of *mestizaje* (race-mixture) and racial democracy purporting colorblindness (Telles and Sue, 2009; Wade, 1997). Scholars have argued that these ideologies are not simply cultural narratives, but serve as a racial project through which the racial/color hierarchies, rooted in legacies of colonialism and racialization, have been both structured and rationalized (Moreno Figueroa and Wade, 2024; Wade, 2024). Within such a context, a perception paradox has emerged. Many individuals perceive ethnoracial discrimination and colorism as a cause of stratification in the region (Silva and Leão, 2012; Telles and Bailey, 2013); yet, this perception coexists with relatively low levels of personal reports of ethnoracial and color discrimination, notions that are deeply intertwined in Latin America given that ethnoracial identification has historically been heavily dependent on skin color and phenotype (Canache et al., 2014; Reyes-Ortiz et al., 2023; Telles and Sue, 2009).

Prior research on ethnoracial and color discrimination and health in Latin America is limited and mixed. While some studies show statistically significant associations with health outcomes (Fattore et al., 2018; Reyes-Ortiz et al., 2023; Santana et al., 2007), others find weak, attenuated, or null associations, particularly when also accounting for class discrimination (Camelo et al., 2018; Mendez et al., 2020; Perreira and Telles, 2014). Given the gap between generalized recognition of color discrimination and personal reports, standard approaches focused on personal experiences may obscure the role of color discrimination as a risk factor for health in this context. National ideologies that discourage recognition of color discrimination may not only shape what is perceived and reported, but also understandings of the relationship between color discrimination and health. Although still understudied compared to personal discrimination, growing evidence suggests that witnessing discrimination against other individuals, also known as vicarious discrimination, is associated with adverse health (Chae et al., 2021; Holloway and Varner, 2023; Moody et al., 2023). Despite the high levels of vicarious discrimination reported in Latin America, little scholarly attention has been paid to the relationship between vicarious color discrimination and health in this context. Considering both personal and vicarious color discrimination may help to uncover whether the narrow focus on personal color discrimination has unduly shaped existing understandings of how color discrimination relates to health.

In this study, I examine the relationship between perceptions of color discrimination and self-rated health in Latin America. I compare multiple attributes of discrimination by examining color and class discrimination, experienced personally and witnessed against others. Class discrimination serves as a salient counterpoint to color because national ideologies have historically privileged class-based explanations of stratification while dismissing the role of color (Degler, 1986; Telles, 2004). This dual comparison provides analytic leverage to investigate how the sociohistorical meaning of the attributes of inequality—in this case, skin color and class status—may shape the relationship between discrimination and health. To do this, I use data from the Project on Ethnicity and Race in Latin America (PERLA). I find that observed associations between discrimination and health vary based on both the target and attribute of discrimination.

## 2 Background

Throughout much of Latin America, darker skin is predictive of worse socioeconomic and health outcomes, at times even more so than racial identification, giving rise to the region being deemed a pigmentocracy (Lipschutz, 1944; Perreira and Telles, 2014; Telles and PERLA, 2014). This racial/color hierarchy was birthed through colonialism, enslavement, and pervasive marginalization of Indigenous and Afrodescendant individuals (ECLAC, 2016; Wade, 2024). These racialized hierarchies based on descent and phenotype created economic and social inequalities that persist to the present day (Telles and PERLA, 2014). However, after independence, national ideologies of *mestizaje,* and racial democracy in Brazil, were used to obscure these hierarchies and construct national identities based on the idea of a racially and culturally mixed society free of racism and racial discrimination (Andrews, 2004; Degler, 1986). While these ideologies assert that race mixture created blurry racial boundaries that constrained racism, scholars argue these ideologies work to legitimize the racial and color-based hierarchy that privileges whiteness and light skin while subordinating darker skin, Indigeneity, and Blackness (Moreno Figueroa and Wade, 2024). Furthermore, these ideologies posit that color-based and ethnoracial inequalities can be explained by colorblind, class-based factors (Harris, 1964; Telles and Sue, 2009).

Yet, the ideologies of *mestizaje* and racial democracy are both contested and incomplete. There is widespread belief that racial discrimination and racism are at least in part to blame for societal inequality (Bastos et al., 2017; Silva and Leão, 2012; Telles and Bailey, 2013). For example, Brazilians with the strongest beliefs in racial democracy counterintuitively most strongly believe that ethnoracial inequalities are produced by structural factors such as racism or discrimination, rather than individualistic explanations such as personal work ethic (Bailey, 2002).

Despite robust evidence that Latin Americans widely perceive racial/color discrimination, rates of reporting personal color discrimination are markedly low (Canache et al., 2014; Dixon, 2019). This discrepancy aligns with a sociohistorical context in which national ideologies can make the reporting of personal discrimination costly. Those who have claimed to be the target of ethnoracial discrimination can themselves be accused of being racist and sowing division (Golash-Boza, 2012; Sue, 2013; Sue and Golash-Boza, 2013; Twine, 1998). Prior work has also suggested that individuals might not perceive or report personal discrimination, as this can cultivate feelings of lack of agency and lower locus of control, given that they seemingly could not prevent themselves from being discriminated against (Lewis et al., 2015).

This gap observed in reports of perceived personal discrimination compared to vicarious discrimination aligns with the personal–group discrepancy, a phenomenon observed in social psychology (Levin et al., 2002; Taylor et al., 1994). Individuals tend to report higher rates of discrimination happening to their in-group members (e.g., Black people in general) than against themselves (e.g., what a Black individual would say they personally experienced). Taylor et al. (1994, 233) argued that “If all minority group members perceive relatively little discrimination directed at them personally, where are the group members who are discriminated against at such a high level that it would warrant the high ratings of group discrimination that emerge so consistently?” This paradox is also highlighted by the fact that many do not report personally knowing others who engage in racist behavior, despite high levels of recognition of color discrimination. For example, in Peru, Sue and Golash-Boza (2013, 1588) concluded that “…although most Peruvians now recognize that racism exists, they do not view themselves or people they know as participating in racism, demonstrating a classic case of ‘racism without racists.”’

Unlike color discrimination, class discrimination was never incongruent with national narratives or identities created through *mestizaje* and racial democracy. According to these narratives, the disadvantages faced by Indigenous and Afrodescendant individuals and those with darker skin were not a result of their skin color or ethnoracial identity. Instead, such disadvantage was explained as resulting from class status (Telles and PERLA, 2014; Telles and Sue, 2009). Consequently, the observed inequality was seen as a historical artifact, not an active contemporary reproduction. For example, according to these ideologies, slavery was seen as a historical reason Afrodescendant individuals were socioeconomically disadvantaged; however, living within a purportedly racially equitable society did not prevent historically marginalized ethnoracial groups from contemporary upward mobility (Andrews, 2004; Telles, 2004). Adages such as “money whitening” also suggested the notion that better treatment comes with increased wealth—even for non-White people or individuals with darker skin (Roth et al., 2022; Schwartzman, 2007). Within such ideologies, class-based explanations thus served to minimize and obscure historically rooted and contemporarily reproduced ethnoracial stratification.

Therefore, according to these national ideologies, class discrimination, unlike color discrimination, was a socially legitimate and expected explanation for inequality (Golash-Boza, 2012; Sue, 2013; Telles, 2004). In line with the predominance of class explanations, class status is often the most common factor to which unfair treatment is attributed in Latin America; rates of reporting personal class discrimination are routinely much higher than those of personal color discrimination (Perreira and Telles, 2014; Silva and Leão, 2012; Telles and PERLA, 2014). Extant research has shown perceptions of class discrimination to be an important risk factor for poor health, one that also attenuates associations between color discrimination and health (Mendez et al., 2020; Perreira and Telles, 2014). These diverging patterns, taken by themselves, would seem to call into question the importance of color-based discrimination in these contexts.

However, national ideologies that privilege class discrimination over color discrimination may complicate individuals’ ability to identify the cause of discriminatory treatment (Canache et al., 2014; Dixon, 2019). Prior work has documented that individuals may experience unfair treatment due to skin color but attribute it to other factors rather than reporting it as color discrimination (Bastos et al., 2017; Dixon, 2019). Given the work of *mestizaje* and racial democracy ideologies to reinterpret racial/color hierarchy through a class-based lens, observed associations between discrimination and health may differ depending on how discrimination is measured. As such, it is vital to examine both the attribute to which individuals assign discriminatory treatment as well as the target of discrimination.

## 3 Data and analytic strategy

### 3.1 Data

To investigate the association between perceptions of discrimination and health in four Latin American countries, I use data from the Project on Ethnicity and Race in Latin America (PERLA). Conducted in 2010, PERLA is uniquely suited for this study as it remains one of the only comparative surveys to offer such extensive questions on color discrimination, particularly both personal and vicarious discrimination, as well as self-rated health in the region. PERLA is a cross-sectional survey conducted using face-to-face interviews with voting-age respondents in Brazil, Colombia, Mexico, and Peru. PERLA selected these countries given their large Indigenous and Afrodescendant populations (Telles and PERLA, 2014). PERLA conducted national surveys in each of the four countries and an Indigenous oversample in Mexico and an Afrodescendant oversample in Colombia. One respondent per household was interviewed and the unit of analysis is the individual. The surveys were administered in Portuguese in Brazil and in Spanish in the three other countries. The number of respondents sampled was approximately 1,000 in Brazil and 1,500 in the other three countries. The analytic sample is restricted to individuals aged 18–65 (*N* = 5,094). Due to sample size limitations, I excluded 174 individuals who did not self-identify as White, Mixed, Indigenous, Black, or Mulato. Finally, I used listwise deletion for the remaining variables to arrive at a final analytic sample of 4,736. All estimates are unweighted and therefore represent associations within this analytic sample, as opposed to population estimates for Latin America.

### 3.2 Variables

The main outcome of interest is self-rated health, which is measured based on the question, “Would you say that your health overall is very good, good, fair, bad, or very bad?” Self-rated health is a robust, widely used measure of well-being that is predictive of morbidity and mortality (Idler and Benyamini, 1997; Jylhä, 2009; Schnittker and Bacak, 2014). In line with a large body of previous work examining poor health as a parsimonious indicator of health status, I created a dichotomous variable for poor health by indicating whether the respondent reported good (good, very good) or poor (fair, bad, very bad) health (de Castro et al., 2020; Fagundes et al., 2022; Perreira and Telles, 2014). The grouping of the “fair” middle category with poor health is consistent with the connotation of the middle category *regular* being more aligned with poor health in Latin America, in contrast to English-language measures of self-rated health in which only the bottom two categories are commonly combined (Camelo et al., 2022; Fagundes et al., 2022; Mendez et al., 2020; Perreira and Telles, 2014).

The main predictors of interest are perceptions of personal and vicarious discrimination based on skin color and class status. These measures are designed to capture self-reported perceptions of discrimination and are also referred to as self-reported discrimination (Williams et al., 2019). The perception of personal color discrimination was based on the following survey item: “In the last five years, have you ever felt discriminated against or been treated badly or unfairly due to: Your skin color.” For personal class discrimination, the respondent was asked the same question with a different discrimination attribute: “Your economic situation.” The perception of vicarious color discrimination was measured based on the following question: “Have you witnessed situations where another person has been discriminated against, treated badly, or unfairly due to: His/her skin color?” For vicarious class discrimination, the respondent was asked the same question with a different attribute: “His/her economic situation?” For each of the discrimination questions, respondents could indicate “many times,” “sometimes,” “a few times,” or “never.” The top three levels of frequency were collapsed to form dichotomous variables measuring whether a respondent reported any level of frequency of discrimination versus no discrimination at all.

Additional covariates included in the analyses are the respondents’ skin color, ethnoracial self-identification, Indigenous parentage, wealth, educational attainment, possession of health insurance, urban/rural residence, gender, age, marital status, and country. Skin color is based on the PERLA Color Palette that ranges from 1 (lightest) to 11 (darkest) (Telles and PERLA, 2014). PERLA interviewers assigned a numerical value to the facial color of the respondent based on this palette. I recoded skin color into a 4-level variable of very light (1, 2), light (3, 4), medium (5, 6), and dark (7–11) in line with previous studies that suggest the relationship between skin color and health outcomes is not linear in the Latin American context (Perreira and Telles, 2014).

Ethnoracial identification with a marginalized group is associated with perceiving ethnoracial discrimination (Canache et al., 2014). Throughout Latin America, skin color and race are often correlated but are not synonymous, and the strength of the association varies by country (Telles and Paschel, 2014). Importantly, decisions on how individuals self-identify can be shaped by political and ideological positions, in addition to, or with little regard for, phenotype (Telles and PERLA, 2014). Therefore, I also control for ethnoracial self-identification based on the question, “You consider yourself… (Spanish) / Your color or race is…? (Portuguese).” I then recoded the responses for standardization across countries to White, Mixed (Mestizo in Spanish-speaking countries/Parda in Brazil), Indigenous, and a joint Black/Mulato category (Negra/Preta/Mulato). The combination of the Black/Mulato category follows Perreira and Telles (2014), who did not find meaningful differences between those who self-identified as Mulato or Black regarding socioeconomic status and health outcomes in PERLA. In Mexico and Peru, respondents were also asked, “Based on your ancestors and customs, you consider yourself to be of what origin?” I also coded those who responded “Nahuatl, Maya, Zapoteco, Mixteco, Quechua, Aymara, De la Amazonia, Otra pueblo indigena” as Indigenous. Indigenous parentage is a binary variable based on self-report that either or both parents were Indigenous.

Socioeconomic status is operationalized using wealth and educational attainment. The wealth measure is an index constructed using principal component analysis of 11 household items (television, refrigerator, telephone, washing machine, vehicle or car, drinking water in the house, bathroom in the house, computer, flat screen TV, internet service, and a gas or electric stove), following Córdova (2009) to account for differences across both urban and rural areas and the various country contexts. The wealth index was normed to have a mean of zero and has a standard deviation of 1.8 with a range of −6.4 to 6.0. Wealth measures are known to be valid alternatives to income-based measures in low-to-middle income nations (Filmer and Pritchett, 2001). Education is measured as the last year of education completed and is a continuous variable ranging from 0 to 18.

Marital status is a binary variable indicating whether the respondent is married/cohabitating or not. Possession of health insurance is also a binary variable indicating whether the respondent reported having any health insurance. Gender and urban setting are binary variables. Age is a continuous variable. In all regression models, I also control for country of residence.

### 3.3 Analytic plan

I begin by presenting summary statistics for the variables of interest in Table 1. Next, I estimate logistic regression models to examine how key demographic factors predict the reporting of perceived discrimination and how these patterns vary by the target (personal versus vicarious) and attribute (color versus class) of discrimination (Table 2). In Table 3, I examine the main question of interest: the relationship between perceived personal color and vicarious color discrimination and poor health using logistic regression models. Model 1 presents the traditional model that is commonly employed to estimate the relationship between personal color discrimination and poor health (Perreira and Telles, 2014). In Model 2, I replace personal color discrimination with the more rarely examined measure of vicarious color discrimination. Finally, to assess the relationship with health when accounting for the other type of discrimination, in Model 3, both personal and vicarious color discrimination are included in the same model.

**Table 1:** Descriptive statistics.

Variable	Value
Poor health (%)	36.7
Perceived discrimination (%)	
Personal color discrimination	19.5
Vicarious color discrimination	59.5
Personal class discrimination	33.0
Vicarious class discrimination	64.5
Skin color (%)	
Very light	6.7
Light	40.0
Medium	36.3
Dark	17.0
Ethnoracial identity (%)	
White	16.1
Mixed	46.8
Indigenous	21.3
Black/*Mulato*	15.8
Parent indigenous	23.4
Socioeconomic status	
Wealth index, mean (SD = 1.8)	0.0
Education (years)	9.6
Healthcare access (%)	
Any health insurance	59.6
Urban	76.1
Demographics (%)	
Female	51.5
Age (years)	37.4
Married/cohabitating	62.1
Country (%)	
Brazil	16.4
Colombia	27.6
Peru	28.8
Mexico	27.2
Observations	4,736

**Table 2:** Odds ratios from logistic regression models predicting perceiving personal and vicarious color and class discrimination.

Variable	(1)	(2)	(3)	(4)
	Personal color	Vicarious color	Personal class	Vicarious class
Skin color (ref. very light)				
Light	0.892 (0.188)	1.041 (0.157)	1.336^+^ (0.202)	1.077 (0.153)
Medium	1.435^+^ (0.272)	1.126 (0.195)	1.386*^∗^* (0.212)	1.079 (0.167)
Dark	2.619*^∗∗∗^* (0.590)	1.591*^∗^* (0.295)	1.760*^∗∗^* (0.321)	1.402^+^ (0.271)
Ethnoracial identity (ref. White)				
Mixed	1.486*^∗∗^* (0.227)	1.055 (0.114)	1.176^+^ (0.114)	1.156 (0.129)
Indigenous	1.695*^∗^* (0.364)	0.970 (0.135)	1.119 (0.159)	1.187 (0.175)
Black/*Mulato*	3.255*^∗∗∗^* (0.664)	1.530*^∗∗^* (0.241)	1.089 (0.172)	1.434*^∗^* (0.234)
Indigenous parent (ref. non-indigenous parent)				
Parent indigenous	1.542*^∗∗∗^* (0.182)	1.347*^∗∗^* (0.131)	1.370*^∗∗∗^* (0.128)	1.239*^∗^* (0.119)
Socioeconomic status				
Wealth index	0.953^+^ (0.027)	1.051^+^ (0.027)	0.896*^∗∗∗^* (0.020)	1.035 (0.027)
Years of education	1.025*^∗^* (0.012)	1.062*^∗∗∗^* (0.010)	1.013 (0.009)	1.067*^∗∗∗^* (0.010)
Demographics				
Female	0.966 (0.069)	0.945 (0.057)	1.052 (0.059)	0.998 (0.061)
Age	0.998 (0.003)	0.993*^∗^* (0.003)	1.002 (0.002)	0.998 (0.002)
Urban	1.346*^∗^* (0.181)	1.523*^∗∗∗^* (0.181)	1.271*^∗^* (0.134)	1.555*^∗∗∗^* (0.180)
Country (ref. Mexico)				
Brazil	0.660*^∗^* (0.117)	0.302*^∗∗∗^* (0.036)	0.639*^∗∗^* (0.093)	0.227*^∗∗∗^* (0.031)
Colombia	0.709^+^ (0.125)	1.114 (0.139)	0.998 (0.139)	0.939 (0.127)
Peru	1.651*^∗∗^* (0.259)	1.353*^∗^* (0.171)	1.220 (0.167)	1.200 (0.162)
Constant	0.061*^∗∗∗^* (0.020)	0.651 (0.171)	0.195*^∗∗∗^* (0.049)	0.710 (0.183)
*n*	4,736	4,736	4,736	4,736

Exponentiated coefficients; Standard errors in parentheses.

^+^*p* < 0.10; ^∗^*p* < 0.05; ^∗∗^*p* < 0.01; ^∗∗∗^*p* < 0.001.

**Table 3:** Odds ratios from logistic regression models for associations between color discrimination and poor health.

Variable	Model 1	Model 2	Model 3
Perceived discrimination (ref. none)			
Any personal color discrimination	1.204 (0.138)		1.120 (0.128)
Any vicarious color discrimination		1.274*^∗∗^* (0.095)	1.241*^∗∗^* (0.092)
Skin color (ref. very light)			
Light	0.960 (0.156)	0.953 (0.158)	0.956 (0.158)
Medium	1.162 (0.206)	1.166 (0.207)	1.161 (0.207)
Dark	1.028 (0.226)	1.035 (0.229)	1.019 (0.226)
Ethnoracial identity (ref. white)			
Mixed	1.169 (0.149)	1.172 (0.149)	1.169 (0.148)
Indigenous	1.061 (0.177)	1.071 (0.178)	1.066 (0.177)
Black/*Mulato*	1.114 (0.203)	1.116 (0.203)	1.100 (0.202)
Indigenous parent (ref. non-indigenous parent)			
Parent indigenous	1.173 (0.114)	1.173 (0.115)	1.165 (0.113)
Socioeconomic status			
Wealth index	0.896*^∗∗∗^* (0.019)	0.892*^∗∗∗^* (0.019)	0.893*^∗∗∗^* (0.019)
Years of education	0.952*^∗∗∗^* (0.010)	0.950*^∗∗∗^* (0.010)	0.950*^∗∗∗^* (0.010)
Healthcare access			
Any health insurance	0.878^+^ (0.068)	0.881 (0.068)	0.882 (0.068)
Demographics			
Female	1.325*^∗∗∗^* (0.084)	1.327*^∗∗∗^* (0.085)	1.327*^∗∗∗^* (0.085)
Age	1.036*^∗∗∗^* (0.003)	1.036*^∗∗∗^* (0.003)	1.036*^∗∗∗^* (0.003)
Urban	0.649*^∗∗∗^* (0.053)	0.639*^∗∗∗^* (0.052)	0.637*^∗∗∗^* (0.052)
Married/Cohabitating	0.955 (0.066)	0.959 (0.067)	0.960 (0.067)
Country (ref. Mexico)			
Brazil	0.620*^∗∗^* (0.106)	0.654*^∗^* (0.111)	0.653*^∗^* (0.111)
Colombia	0.535*^∗∗∗^* (0.067)	0.527*^∗∗∗^* (0.066)	0.530*^∗∗∗^* (0.067)
Peru	1.898*^∗∗∗^* (0.227)	1.900*^∗∗∗^* (0.232)	1.886*^∗∗∗^* (0.228)
Constant	0.260*^∗∗∗^* (0.066)	0.237*^∗∗∗^* (0.061)	0.238*^∗∗∗^* (0.061)
*n*	4,736	4,736	4,736

Exponentiated coefficients; Standard errors in parentheses.

^+^*p* < 0.10; ^∗^*p* < 0.05; ^∗∗^*p* < 0.01; ^∗∗∗^*p* < 0.001.

I then repeat the analysis above, but for perceived class discrimination as a comparator to perceived color discrimination to assess whether the results based on the target (personal vs. vicarious) hold across attributes. In Table 4, I show the relationship between poor health and personal class discrimination, then vicarious class discrimination, and both simultaneously (Models 1–3). Finally, in Models 4–6 of Table 4, I estimate the association with poor health for personal color and personal class discrimination, vicarious color and vicarious class discrimination, and all four types of discrimination simultaneously.

**Table 4:** Odds ratios from logistic regression models for associations between discrimination and poor health.

Variable	Model 1	Model 2	Model 3	Model 4	Model 5	Model 6
Perceived class discrimination (ref. none)						
Any personal class discrimination	1.338*^∗∗∗^* (0.103)		1.321*^∗∗∗^* (0.104)	1.327*^∗∗∗^* (0.105)		1.325*^∗∗∗^* (0.111)
Any vicarious class discrimination		1.153^+^ (0.090)	1.037 (0.082)		0.970 (0.090)	0.881 (0.084)
Perceived color discrimination (ref. none)						
Any personal color discrimination				1.020 (0.126)		0.971 (0.120)
Any vicarious color discrimination					1.299*^∗∗^* (0.115)	1.288*^∗∗^* (0.118)
Skin color (ref. very light)						
Light	0.944 (0.155)	0.954 (0.157)	0.944 (0.155)	0.945 (0.154)	0.954 (0.158)	0.944 (0.155)
Medium	1.154 (0.205)	1.169 (0.208)	1.154 (0.205)	1.153 (0.206)	1.166 (0.207)	1.153 (0.206)
Dark	1.027 (0.226)	1.049 (0.231)	1.026 (0.226)	1.024 (0.227)	1.035 (0.230)	1.017 (0.226)
Ethnoracial identity (ref. white)						
Mixed	1.164 (0.148)	1.170 (0.148)	1.163 (0.148)	1.163 (0.148)	1.173 (0.149)	1.166 (0.148)
Indigenous	1.065 (0.178)	1.064 (0.177)	1.064 (0.178)	1.064 (0.177)	1.072 (0.178)	1.073 (0.178)
Black/*Mulato*	1.138 (0.207)	1.130 (0.204)	1.135 (0.207)	1.135 (0.207)	1.116 (0.204)	1.125 (0.207)
Indigenous parent (ref. non-indigenous parent)						
Parent indigenous	1.165 (0.115)	1.183^+^ (0.116)	1.165 (0.115)	1.164 (0.114)	1.173 (0.115)	1.157 (0.113)
Socioeconomic status						
Wealth index	0.901*^∗∗∗^* (0.019)	0.894*^∗∗∗^* (0.019)	0.900*^∗∗∗^* (0.020)	0.900*^∗∗∗^* (0.019)	0.892*^∗∗∗^* (0.019)	0.898*^∗∗∗^* (0.019)
Years of education	0.952*^∗∗∗^* (0.010)	0.951*^∗∗∗^* (0.010)	0.952*^∗∗∗^* (0.010)	0.952*^∗∗∗^* (0.010)	0.950*^∗∗∗^* (0.010)	0.950*^∗∗∗^* (0.010)
Healthcare access						
Any health insurance	0.885 (0.068)	0.878^+^ (0.068)	0.886 (0.068)	0.885 (0.068)	0.881 (0.068)	0.888 (0.069)
Demographics						
Female	1.319*^∗∗∗^* (0.083)	1.323*^∗∗∗^* (0.084)	1.319*^∗∗∗^* (0.084)	1.319*^∗∗∗^* (0.084)	1.327*^∗∗∗^* (0.084)	1.323*^∗∗∗^* (0.084)
Age	1.036*^∗∗∗^* (0.003)	1.036*^∗∗∗^* (0.003)	1.036*^∗∗∗^* (0.003)	1.036*^∗∗∗^* (0.003)	1.036*^∗∗∗^* (0.003)	1.036*^∗∗∗^* (0.003)
Urban	0.644*^∗∗∗^* (0.053)	0.646*^∗∗∗^* (0.052)	0.643*^∗∗∗^* (0.053)	0.644*^∗∗∗^* (0.052)	0.639*^∗∗∗^* (0.052)	0.637*^∗∗∗^* (0.052)
Married/Cohabitating	0.956 (0.066)	0.953 (0.066)	0.956 (0.066)	0.956 (0.066)	0.959 (0.067)	0.962 (0.067)
Country (ref. Mexico)						
Brazil	0.634*^∗∗^* (0.109)	0.644*^∗∗^* (0.110)	0.641*^∗∗^* (0.109)	0.634*^∗∗^* (0.109)	0.651*^∗^* (0.111)	0.648*^∗^* (0.110)
Colombia	0.530*^∗∗∗^* (0.066)	0.532*^∗∗∗^* (0.067)	0.530*^∗∗∗^* (0.066)	0.530*^∗∗∗^* (0.067)	0.527*^∗∗∗^* (0.066)	0.525*^∗∗∗^* (0.066)
Peru	1.910*^∗∗∗^* (0.233)	1.916*^∗∗∗^* (0.232)	1.908*^∗∗∗^* (0.232)	1.907*^∗∗∗^* (0.230)	1.900*^∗∗∗^* (0.233)	1.897*^∗∗∗^* (0.231)
Constant	0.248*^∗∗∗^* (0.064)	0.246*^∗∗∗^* (0.064)	0.245*^∗∗∗^* (0.063)	0.248*^∗∗∗^* (0.063)	0.238*^∗∗∗^* (0.062)	0.237*^∗∗∗^* (0.061)
*n*	4,736	4,736	4,736	4,736	4,736	4,736

Exponentiated coefficients; Standard errors in parentheses.

^+^*p* < 0.10; ^∗^*p* < 0.05; ^∗∗^*p* < 0.01; ^∗∗∗^*p* < 0.001.

## 4 Results

Table 1 displays descriptive statistics for the sample. Regarding health, 36.7% of the sample reports poor health. Personal color discrimination is perceived by 19.5% of individuals. In comparison, more than three times as many individuals (59.5%) report they have witnessed someone else being discriminated against due to skin color (vicarious color discrimination). Personal class discrimination is considerably more common than personal color discrimination, with 33.0% of individuals perceiving they have been discriminated against personally due to class status. The percentage of those perceiving vicarious class discrimination (64.5%) is almost twice as high as personal class discrimination, but not much higher than the rate for vicarious color discrimination. Most respondents have either light (40.0%) or medium skin color (36.3%), while dark (17.0%) and very light (6.7%) skin color are less common. Ethnoracial self-identification as Mixed is the most common response (46.8%), followed by Indigenous (21.3%), then White (16.1%), and lastly Black/Mulato (15.8%), with 23.4% also reporting Indigenous parentage. The majority of respondents reside in urban areas, have health insurance, and are married or cohabitating. Respondents, on average, have 9.6 years of education and a normed wealth index score of approximately zero (SD = 1.8).

Table 2 displays estimates from logistic regression models assessing the relationship between key demographic factors and perceiving personal color discrimination (Model 1) and vicarious color discrimination (Model 2), personal class discrimination (Model 3), and vicarious class discrimination (Model 4), as compared to not perceiving the respective type of discrimination. The results show a different pattern in the factors associated with perceiving personal discrimination. Ethnoracial identification most consistently predicts perceiving personal color discrimination; however, skin color more consistently predicts perceiving personal class discrimination, when adjusting for other factors. The patterns for skin color and ethnoracial identification are fairly consistent between vicarious color discrimination and vicarious class discrimination. In terms of socioeconomic status, more years of education is positively associated with perceiving all but personal class discrimination. On the other hand, greater wealth is associated with significantly lower odds of reporting personal class discrimination, while for the other measures of discrimination it is marginally or not statistically significant.

Next, Table 3 displays odds ratios for the relationships between perceived personal and vicarious color discrimination and poor health. All models were adjusted for skin color, ethnoracial identification, Indigenous parentage, wealth, education, health insurance, gender, age, urban residence, marital status, and country of residence. In Model 1, the association between personal color discrimination and poor health is not statistically significant, holding all else equal. However, those who perceive vicarious color discrimination (Model 2) have 27% higher odds of reporting poor health compared to those who do not perceive vicarious color discrimination (OR: 1.27, *p* < 0.01). In Model 3, the association between vicarious color discrimination and poor health remains statistically significant when both personal and vicarious color discrimination are included. Those who perceive vicarious color discrimination have 24% higher odds of reporting poor health compared to those who do not perceive vicarious color discrimination (OR: 1.24, *p* < 0.01).

Next, in Table 4, I examine the relationship between personal and vicarious class discrimination and poor health. In Table 4, Model 1 shows that those who perceive personal class discrimination have 34% higher odds of reporting poor health than those who do not perceive personal class discrimination (OR: 1.34, *p* < 0.001). This finding contrasts with the findings for personal color discrimination, in which there is not a statistically significant association between personal color discrimination and poor health. For Model 2, vicarious class discrimination is marginally associated with poor health (OR: 1.15, *p* < 0.10). When including both personal class discrimination and vicarious class discrimination, Model 3 shows personal class discrimination has a statistically significant association with poor health (OR: 1.32, *p* < 0.001). However, the relationship between vicarious class discrimination and poor health is not statistically significant.

I introduce my final line of comparative investigation in Models 4–6. Model 4 displays odds ratios from a logistic regression in which personal color and personal class discrimination predict poor health. When adjusting for personal color discrimination, personal class discrimination remains associated with poor health, while the relationship between personal color discrimination and poor health is not statistically significant. Those who perceive personal class discrimination have 33% higher odds of reporting poor health than those who do not perceive personal class discrimination (OR: 1.33, *p* < 0.001). Model 5 shows that vicarious color discrimination is associated with poor health (OR: 1.30, *p* < 0.01), while vicarious class discrimination does not show a statistically significant association with poor health when controlling for vicarious color discrimination. Finally, in Model 6, I include all four measures of discrimination in the same model. When adjusting for the other types of discrimination, both vicarious color discrimination and personal class discrimination remain associated with poor health at a statistically significant level. Those who witness color discrimination have 29% higher odds of reporting poor health (OR: 1.29, *p* < 0.01), and those perceiving that they have experienced class discrimination have 33% higher odds of reporting poor health (OR: 1.33, *p* < 0.001) when holding all else constant. Personal color discrimination and vicarious class discrimination do not show statistically significant associations.

## 5 Discussion

The findings of this study contribute to the broader literature on perceptions of discrimination and health by demonstrating how measurement approaches shape observed associations with health. Leveraging PERLA’s unique set of extensive measures of discrimination, I find that vicarious color discrimination and personal class discrimination demonstrate the most consistent associations with poor health, while there is no evidence of a statistically significant association with personal color discrimination. Taken together, these findings suggest that associations between perceived discrimination and health must be understood in light of the Latin American racial order in which *mestizaje* and racial democracy work to define which explanations of inequality and unfair treatment are deemed legitimate and reportable.

The results demonstrate that color discrimination’s association with health varies based on the target of the discrimination. Personal color discrimination did not show a statistically significant association with poor health, which is consistent with prior studies that have found weak or attenuated associations (Mendez et al., 2020; Perreira and Telles, 2014). In contrast, vicarious color discrimination showed a statistically significant association with increased odds of reporting poor health even when accounting for the other measures of discrimination. This finding extends growing evidence from the U.S. that vicarious discrimination is a risk factor for health (Chae et al., 2021; Dominguez et al., 2008; Holloway and Varner, 2023; Moody et al., 2023; Wofford et al., 2019). Given that the majority of individuals report vicarious color discrimination, the health implications of living in a pigmentocracy may be more far-reaching than previously understood and not limited to the targets of color-based discrimination.

The differential finding for personal and vicarious color discrimination may in part reflect national ideologies shaping recognition and reporting of discrimination. National ideologies encourage citizens not to interpret racial and color discrimination as the cause of unfair treatment but instead hold other forces, such as classism, responsible or attribute the treatment to personal failure (Harris and Kottak, 1963; Pierson, 1942). Correspondingly, personal color discrimination was the least common type of discrimination reported. This is consistent with other studies finding low levels of prevalence (Canache et al., 2014; Dixon, 2019; Reyes-Ortiz et al., 2023). Prior work has argued that individuals may attribute unfair treatment they experience to individual characteristics rather than to a social category, thus not perceiving unfair treatment as discrimination (Machado et al., 2021; Silva and Leão, 2012). For example, Bastos et al. (2017, 304) argued that their Brazilian respondents’ conceptualization of mistreatment and discrimination is distinct, thereby structuring what is reported as discrimination. This conceptualization is shaped by “… the ideologies that individuals hold—particularly the extent to which they view inequalities as stemming from individual characteristics or broader social structures.”

In such a sociohistorical context with pervasive color-based stratification, reporting vicarious color discrimination may be less psychologically and socially costly than reporting personal color discrimination (Lewis et al., 2015; Sue and Golash-Boza, 2013; Twine, 1998). Close to 60% of respondents report vicarious color discrimination, as compared to about 20% who report personal color discrimination; the high prevalence of vicarious discrimination may serve to make it more socially permissible. This pattern of reporting may also enable individuals to acknowledge clear ethnoracial disparities while minimizing some of the social risks of challenging *mestizaje* ideologies (Sue and Golash-Boza, 2013). The high prevalence of reports of vicarious color discrimination relative to personal color discrimination may also reflect an awareness of racial hierarchies and inequality rooted in colonialism, slavery, and racialization rather than solely the perception of individual-level interactions (ECLAC, 2016; OHCHR, 2023; PAHO, 2019; Wispelwey et al., 2023).

While this study examines measures of interpersonal discrimination, the findings must be considered in light of the specific sociohistorical legacies marked by structural racism and racial hierarchies in which racism and discrimination operate as structural determinants of health (OHCHR, 2023; Williams et al., 2019; Wispelwey et al., 2023). Those who witness discrimination may recognize that they themselves are susceptible to being discriminated against, which in turn can have negative mental and physical health effects (Truong et al., 2016). Given the relatively blurry and fluid racial boundaries in Latin America, and the fact that skin color is often contextualized as relative (e.g., relative color of siblings within families), this potential susceptibility may be particularly salient (Hordge-Freeman, 2015; Telles and Paschel, 2014). Living in such a context may lead to anticipatory stress, hypervigilance, and rumination, which may contribute to weathering and allostatic load due to the stress of being constantly on guard against potential discrimination (Himmelstein et al., 2015; Williams et al., 2019).

The diverging findings for class discrimination reveal that the association between the target of discrimination and health varies by the attribute of discrimination. Personal class discrimination was both more commonly reported than personal color discrimination and associated with poor health in line with prior research (Mendez et al., 2020; Perreira and Telles, 2014). This pattern aligns with the work of national ideologies to legitimate class-based explanations while obscuring racial/color-based ones (Moreno Figueroa and Wade, 2024; Wade, 2024). In a context in which color and class are historically intertwined and hard to disentangle, prior work has identified the difficulty individuals may have in assigning a cause of discrimination such as class or color discrimination (Dixon, 2019; Merritt et al., 2006). Notably, the results show skin color was more consistently associated with reporting personal class discrimination than personal color discrimination, a pattern consistent with some unfair treatment based on color being attributed to class factors (Dixon, 2019). If this study had solely examined perceptions of personal discrimination, the pattern of results would mirror previous work showing that class discrimination was associated with poor health, whereas color discrimination was not (Mendez et al., 2020; Perreira and Telles, 2014). The relationship found for vicarious color discrimination highlights how such approaches have led to an incomplete understanding of perceived discrimination and health in this sociohistorical context.

Conversely, the association between vicarious class discrimination and health is not statistically significant. This suggests vicarious discrimination is not a universally more consistent risk factor for poor health than personal discrimination in this context. Notably, vicarious color discrimination and vicarious class discrimination were reported at similar rates and the factors predicting both were largely consistent. This similarity for both types of vicarious discrimination suggests a generalized awareness of the broader inequality and the ways both class and color structure life outcomes. Taken together, the divergent findings for vicarious color and vicarious class discrimination suggest vicarious discrimination measures may reflect a broader recognition of societal inequality; on the other hand, personal reports may more strongly align with national ideologies. These diverging patterns highlight the need for scholars to pay closer attention to measurement approaches.

This study has several limitations. First, with cross-sectional data, it is not possible to establish a causal relationship between perceiving discrimination and health. However, prior reviews of longitudinal studies provide additional evidence of the association between discrimination and worse health (Lewis et al., 2015; Paradies et al., 2015). Future work assessing longitudinal associations between personal and vicarious color and class discrimination is needed. Second, future work should more closely examine the patterns found in this study within country-specific contexts. Although Latin American countries share broad similarities, each country possesses a unique sociohistorical context with differing manifestations of the national ideologies as well as patterns of stratification and inequality (Loveman, 2014; Telles and Paschel, 2014; Telles and PERLA, 2014). A third limitation is that although PERLA remains one of the only datasets in the region to examine personal and vicarious color discrimination and self-rated health, the data were collected in 2010. Given the evolving discourse on race, colorism, and discrimination throughout the region, future studies should investigate whether the reporting patterns and observed associations found in this study persist in the present context.

Lastly, the measure for personal discrimination is bounded by the time frame of “the last five years,” while the time frame for vicarious discrimination is unspecified. Prior work suggests chronic discrimination, particularly the accumulation of discrimination over the life course, may have especially deleterious effects on health (Lewis et al., 2015; Williams et al., 2019). As a result, the statistically significant association between vicarious color discrimination and health, compared to the null finding for personal color discrimination, may in part arise due to the longer exposure period implied by the vicarious discrimination measures. However, the existence of a statistically significant association for personal class discrimination but not for vicarious class discrimination calls this explanation into question. If the discrepancy were simply due to the time difference, one would expect to find the same pattern for class discrimination, with vicarious class discrimination also being associated with health. The actual pattern of results suggests that this difference is not solely due to the time reference of the question. It will be important for future work to examine both personal and vicarious discrimination through longitudinal research.

## 6 Conclusion

By examining both personal and vicarious color discrimination, this study expands the more limited role color discrimination has occupied in understanding perceived discrimination as a risk factor for health in Latin America. Reframing discrimination’s observed relationship to health as shaped by the target reveals an unexamined complexity in understanding colorism and its implications. The divergent findings for color and class discrimination, with vicarious color discrimination and personal class discrimination showing the most consistent associations with poor health, demonstrate how overlooking the sociohistorical meaning of the attribute and the target of discrimination can lead to incomplete conclusions. As such, efforts to examine stratification and inequality must seriously consider how individuals interpret, understand, and report societal processes and how this meaning-making shapes scholarly conclusions.

## Data Availability

Publicly available datasets were analyzed in this study. Data used in this study can be requested from https://perla.soc.ucsb.edu/perla-data-sets.
